# Trends in the use of electrical cardioversion for atrial fibrillation: influence of major trials and guidelines on clinical practice

**DOI:** 10.1186/1471-2261-12-42

**Published:** 2012-06-18

**Authors:** Josep M Alegret, Xavier Viñolas, César Romero-Menor, Silvia Pons, Roger Villuendas, Naiara Calvo, Jordi Pérez-Rodon, Xavier Sabaté

**Affiliations:** 1 Secció de Cardiologia, Hospital Universitari de Sant Joan, Institut d’Investigacions Sanitàries Pere Virgili, Universitat Rovira i Virgili, C/Dr. Laporte, s/n, Reus, 43205, Spain; 2 Hospital de la Sta. Creu i Sant Pau, Barcelona, Spain; 3 Parc Sanitari Sant Joan de Déu, Sant Boi de Llobregat, Spain; 4 Hospital de Barcelona, Barcelona, Spain; 5 Hospital Germans Trias i Pujol, Badalona, Spain; 6 Hospital Clínic, Barcelona, Spain; 7 Hospital Vall d’Hebron, Barcelona, Spain; 8 Hospital de Bellvitge, Hospitalet de Llobregat, Spain

**Keywords:** Atrial fibrillation, Electrical cardioversion, Rhythm control, Rate control, Follow-up

## Abstract

**Background:**

The purpose of the present study was to assess the trends in the use of ECV following published studies that had compared rhythm and rate control strategies on atrial fibrillation (AF), and the recommendations included in the current clinical practice guidelines.

**Methods:**

The REVERCAT is a population-based assessment of the use of electrical cardioversion (ECV) in treating persistent AF in Catalonia (Spain). The initial survey was conducted in 2003 and the follow-up in 2010.

**Results:**

We observed a decrease of 9% in the absolute numbers of ECV performed (436 in 2003 *vs.* 397 in 2010). This is equivalent to 27% when considering population increases over this period. The patients treated with ECV in 2010 were younger, had a lower prevalence of previous embolism, a higher prevalence of diabetes, and increased body weight. Underlying heart disease factors indicated, in 2010, a higher proportion of NYHA ≥ II and left ventricular ejection fraction <30%. We observed a reduction in the number of ECV performed in 16 of the 27 (67%) participating hospitals. However, there was an increase of 14% in the number of procedures performed in tertiary hospitals, and was related to the increasing use of ECV as a bridge to AF ablation. Considering the initial number of patients treated with ECV, the rate of sinus rhythm at 3 months was almost unchanged (58% in 2003 *vs.* 57% in 2010; p = 0.9) despite the greater use of biphasic energy in 2010 and a similar prescription of anti-arrhythmic drugs.

**Conclusions:**

Although we observed a decrease in the number of ECVs performed over the 7 year period between the two studies, this technique remains a common option for treating patients with persistent AF. The change in the characteristics of candidate patients did not translate into better outcomes.

## Background

The REVERCAT study (*REgistre sobre la cardioVERSió elèctrica a CATalunya*; Registry of Electrical Cardioversion in Catalonia) is a multi-center study involving 27 participating hospitals. The recording of characteristics of electrical cardioversion (ECV) was intended to evaluate the use of the technique in patients with atrial fibrillation (AF) in current clinical practice in Catalonia (Spain). The initial study was conducted at the start of 2003. The current re-evaluation was conducted early in 2010 and, over these past 7 years, there have been a few studies published, and subsequent guidelines generated, which described the lack of a clear benefit of rhythm control *vs.* rate control strategies in patients with AF [1-3]. The purpose of the present study was to compare the frequency and characteristics of patients treated with ECV between the years 2003 and 2010; the objective being to assess the impact of major clinical trials and recommendations included in the current clinical practice guidelines.

## Methods

The REVERCAT study was set-up to record, prospectively, all patients with persistent AF who were considered candidates for ECV. There were 27 participating hospitals which are representative of the whole of Catalonia (an Autonomous Community in NE Spain) (Table 1).

**Table 1:** List of investigators and centers participating in the REVERCAT study

**Clinical Investigator**^**a**^	**Affiliation**
X Sabaté	H.U. de Bellvitge, Hospitalet de Llobregat
R Serrat	H del Mar
A Sualís	H de Mataró
F Planas	H. de Badalona
G Vazquez	H. Comarcal de la Selva, Blanes
J Escudero	H Mútua de Terrassa
X Abardia/C Barberà	Clínica Ponent, Lleida
L Guillamon	H. Parc Taulí, Sabadell
L Mont/N Calvo	H. Clínic, Barcelona
J Sadurní	H. General de Vic
S Pons	H de Barcelona
A Descalzi	H. Comarcal Alt Penedés, Vilafranca del Penedés
N Batalla	H. Sagrat Cor, Barcelona
E Sanz/S Serrano	H.U. Joan XXIII, Tarragona
J Pérez-Rodon	H.G.U. Vall d’ Hebrón, Barcelona
E Rodríguez-Font	H. Sta Creu i Sant Pau, Barcelona
F Freire	H. de Palamós, Palamós
M Vilaseca	H de Calella, Calella
C Romero	H. Sant Boi de Llobregat
I Duran	H.U. Sant Joan de Reus
J Tomàs	H.U. Arnau de Vilanova, Lleida
I Lechuga	H. Verge de la Cinta, Tortosa
R Villuendas	H. Germans Trias i Pujol, Badalona
A Jaber	H. de Terrassa
I Romeo	H. d’Igualada
R Canals	H. Comarcal de Mollet
M Paz	H de Figueras

^a^Collaborators in Pubmed.

The area of Catalonia is 31,930 km^2^ and the population was 6,506,000 inhabitants in 2003 and 7,512,000 inhabitants in 2010, all of whom have the right to health-care provision under the publicly-funded National Health Service. The hospitals participating in the present study attend to approximately 90% of this population. The initial registry was set-up between 1^st^ February and 30^th^ October 2003. The present study was conducted between 1^st^ February and 30^th^ October 2010, the purpose being to assess the changes in the use of ECV in clinical practice in Catalonia. The patients included in the study to have ECV applied were all those who met the criteria of being ≥18 years of age, with AF duration >7 days, and with no precipitating conditions including hyperthyroidism, fever, cardiac surgery and pericarditis. Successful ECV was considered when sinus rhythm (SR) was achieved, and excluded patients with immediate relapse. A clinical and ECG follow-up was performed at 3 months post-ECV. Patients were considered to have maintained SR at 3 months if there had not been a relapse of persistent AF and, as well, the ECG at 3 months of follow-up showed SR. The information recorded included clinical data, treatment, echocardiography data, and procedure variables. We compared all these variables in the two surveys conducted 7 years apart. The principal investigator in each hospital was the same in both surveys in 21 of the 27 participating hospitals.

The study received approval from the Institutional Review Boards (Clinical Ethics Committee) of each participating hospital on the understanding that the data were coded on entry into the registry and that patient privacy was respected. Written informed consent was obtained from the patient for publication of this report.

### Statistical analyses

Qualitative variables are expressed in percentages, and the differences assessed using the chi-squared test. Quantitative variables are presented as means ± standard deviation (SD) and the differences between means evaluated using the Student *t*-test. Statistical significance was accepted at p values <0.05. All analyses were performed with the SPSS statistical software package (version 18).

## Results

There were 397 ECVs performed in 2010 compared to 436 in 2003. These were consecutive patients meeting the inclusion criteria and having ECV performed over the same period of the year-of-study (February to October). This represents a 9% reduction in the number of procedures which, when taking into account the increase in the catchment population between 2003 and 2010, represents a reduction of 27%.

The clinical characteristics are summarized in Table 2. The patients treated with ECV in 2010 had a lower mean age, a lower prevalence of prior embolism, a higher prevalence of diabetes and a higher mean body weight compared to those treated with ECV in 2003.

**Table 2:** **Comparisons of the clinical and echocardiographic characteristics of patients included in the two surveys (2003*versus*2010) of electrical cardioversion**

**Characteristic**	**2003 survey (n = 436)**	**2010 survey (n = 397)**	**P**
	**Mean ± SD or n (%)**	**Mean ± SD or n (%)**	
Age; years	65 ± 11	64 ± 11	0.03
Age; >70 years	170 (39)	119 (30)	0.008
Male gender	296 (68)	285 (72)	0.19
Weight; kg	78.2 ± 13	80.9 ± 14	0.01
Height; cm	167 ± 9	168 ± 9	0.14
Body surface area; m^2^	1.86 ± 0.19	1.90 ± 0.19	0.006
Hypertension	222 (51)	222 (56)	0.14
Diabetes mellitus	57 (13)	75 (19)	0.04
Previous embolism	46 (12)	29 (7)	0.02
NYHA Class ≥ II	135 (31)	175 (44)	0.0001
Left atrial size	45.4±6.3	45.4±6.8	0.95
Left atrial dilatation (>50mm)	61 (14)	67 (17)	0.42
Left ventricular hypertrophy	113 (26)	119 (30)	0.21
LVEF (%)	58 ± 14	56 ± 14	0.02
LVEF <30%	8 (2)	19 (5)	0.05
Previous electrical cardioversion	65 (15)	87 (22)	0.009
Duration of AF >1 year	48 (11)	59 (15)	0.35
Anti-arrhythmic drugs	327(78)	296 (76)	0.39
Amiodarone:	268 (64)	247 (62)	
Ic:	39 (9)	32 (9)	
Sotalol:	9 (2)	5 (1)	
Others:	11 (3)	12 (3)	
ACE inhibitor/ARB	197 (45)	197 (50)	0.12

*AF: atrial fibrillation; *LVEF*: left ventricular ejection fraction; *Ic*: flecainide, propafenone; *ACE*: angiotensin-converting enzyme; *ARB*: angiotensin receptor blockers.

With respect to the underlying heart disease, in 2010 we observed a higher proportion of NYHA ≥ II, lower mean ejection fraction, and a higher prevalence of EF <30% than in 2003.

There was a reduction in the numbers of patients in whom ECV was applied in 16 of 27 (67%) participating hospitals. However, the distributions of the treatment indicated a higher number of ECV procedures in tertiary-care hospitals (mainly university-associated) compared to district general hospitals between 2003 and 2010. In 2003 the district hospitals accounted for 57% of procedures compared to 43% in the tertiary hospitals. In 2010 the district hospitals performed 47% of the procedures compared to 53% in tertiary hospitals (p = 0.004). Between 2003 and 2010, we observed an absolute decrease of 23% (p = 0.004) in the number of ECVs performed in district hospitals (247 in 2003 *vs.* 189 in 2010). Conversely, there was an absolute increase in the use of ECV in tertiary hospitals of 14% (p = 0.004) in 2010 (189 in 2003 *vs.* 208 in 2010). There were 7 patients in 2003 in whom ECV was applied as a bridge to AF ablation. In 2010 the number increased to 36; 3 centers performing AF ablations in 2003, and 5 centers in 2010. Overall, the numbers of AF ablations performed in the two periods of the study were 30 in 2003 and 129 in 2010.

The ECV success rates were similar in 2003 and 2010 (86% in 2003 *vs.* 89% in 2010; p = 0.21), despite the more frequent use of biphasic energy in 2010 (16% in 2003 *vs.* 88% in 2010; p = 0.0001) (Table 3).

**Table 3:** **Electrical cardioversion procedure characteristics in both surveys (2003*versus*2010)**

**Characteristic**	**2003 survey (n = 436)**	**2010 survey (n = 397)**	**P**
	**Mean ± SD or n (%)**	**Mean ± SD or n (%)**	
Use of biphasic energy	70 (16)	349 (88)	<0.001
Successful ECV (overall)	374 (86)	355 (89)	0.21
- Monophasic energy	313 (85)	42 (87)	0.69
- Biphasic energy	61 (89)	313 (90)	0.90
Number of shocks (overall)	1.73 ± 0.9	1.5 ± 0.9	0.0001
- Monophasic shocks	1.76 ± 0.9	1.70 ± 0.83	0.90
- Biphasic shocks	1.61 ± 0.86	1.45 ± 0.78	0.07
Energy delivered (overall) (J)	239 ± 90	165 ± 63	0.0001
- Monophasic shocks	253 ± 77	234 ± 81	0.11
- Biphasic shocks	166 ± 95	154 ± 51	0.48

*ECV: electrical cardioversion; *J*: Joules.

The use of anti-arrhythmic drugs pre-ECV and at discharge from hospital was also similar in both surveys (pre-ECV 73% in 2003 *vs.* 68% in 2010; p = 0.26; at discharge 78% in 2003 *vs.* 76% in 2010; p = 0.39). Amiodarone was the preferred drug in both studies. It was used in 64% of patients in 2003 *vs.* 62% of patients in 2010 (p = 0.62), independently of underlying left ventricular ejection fraction. Dronedarone was not available in Spain until the end of the 2010 study.

With respect to anticoagulation treatment, the conventional use of coumarins at least 3 weeks pre- and 1 month post-ECV was the most common pattern (2003 59% *vs.* 2010 61%; p = 0.75). The rate of patients requiring chronic anticoagulant therapy prior to the decision to perform ECV was similar (27% in 2003 *vs.* 30% in 2010; p = 0.67), whereas the use of transesophageal echocardiography with short patterns of anticoagulation (5% in 2003 *vs.* 7% in 2010; p = 0.65) and other patterns (9% in 2003 *vs.* 2% in 2010, p = 0.28) were very low in both surveys.

The rate of SR was similar in both surveys at 3 months of follow-up (67% in 2003 *vs.* 64% in 2010; p = 0.21). If we considered the patients with and without anti-arrhythmic drugs at discharge from hospital, the rates of SR at 3 months were also similar. The patients receiving anti-arrhythmic medications in 2003 accounted for 70% of the total compared to 68% in 2010 (p = 0.30). Patients not receiving anti-arrhythmic medications in 2003 accounted for 58% of the total compared to 55% in 2010 (p = 0.59). Of those treated with ECV, the rate of patients in whom the SR was restored and maintained at 3 months was almost unchanged in the two periods of the study (58% in 2003 *vs.* 57% in 2010; p = 0.9).

## Discussion

To the best of our knowledge this is the first population-based study that analyzed the treatment of AF over time, specifically the use of ECV. Also, this was an opportunity to observe the impact in clinical practice of new evidence reflected in guideline recommendations. We observed that between 2003 and 2010 there was a reduction in the number of ECVs performed and a change in the characteristics of patients treated i.e. this finding may be related to a lower use of the rhythm control strategy compared to rate control in the population with the current characteristics . However, ECV continues to be used frequently in clinical practice.

The AFFIRM and RACE trials [1,2] demonstrated no differences in terms of morbido-mortality when comparing rate *vs.* rhythm control strategies in patients with AF. Also the AF-CHF trial [4], focusing on patients with heart failure or left ventricular dysfunction, observed no difference in cardiovascular mortality. Prior to this evidence, it was accepted in clinical practice that the relief of symptoms as well as prevention of embolism and avoidance of cardiomyopathy (theoretically adding to the maintenance of SR) could be reasons for restoration of SR. These consensus opinions are reflected in the guidelines from the ESC/AHA/ACC 2001 [5]. Subsequent to these publications, the concept became accepted that anticoagulation should not be stopped despite the restoration of SR in patients with criteria for anticoagulation. Thus, in the 2006 ESC/AHA/ACC guidelines, the avoidance of long-term anticoagulation was not considered a reason for ECV [3]. The recent ESC 2010 guidelines [6] were published in the course of the conduct of the present study, as were the ACCF/AHA/HRS 2011 guidelines [7] which were published soon after the study’s completion. These guidelines support anticoagulation maintenance (if the patient fulfills the criteria for anticoagulation) despite the patient achieving SR. Thus, guidelines limit the recommendations for ECV to patients with clear symptoms related to AF, without any intention to reduce morbido-mortality. Theoretically, this would imply a reduction in the number of ECVs performed. However, there is scant information regarding the influence of these guidelines on the use of ECV [8] and, perhaps more importantly, the characteristics of patients who are candidates for treatment with ECV. Our initial survey was begun in early 2003, a short time after the publication of the AFFIRM and RACE studies in December 2002, the findings of which are consistent with our findings in 2010. Although we observed a considerable decrease in the number of ECVs performed, this technique remains a common option for treating patients with AF. This may be related to the impression that, when the data from these trials are analyzed according to the patient’s actual rhythm, the benefit of SR over AF becomes apparent [9]. A more profound analysis of AFFIRM study reveals several limitations that preclude the assumption that attempting to restore SR is not worthwhile i.e. the study had a low percentage of SR patients in the rhythm control group, low efficacy and increased risk of death of AAD, a high percentage of SR patients in the rate control group, lower use of beta-blockers in rhythm control group, and exclusion of patients with severe symptoms who would, potentially, most benefit from SR. Strictly, the study should not be interpreted as a comparison of SR *vs.* AF and, indeed, may reflect the ineffectiveness of the rhythm control methods used.

From our results we may deduce a change in the indications for ECV. Current patients are more symptomatic for heart failure, and with higher disease burden. We also noted that in the 2003 survey the patients were less symptomatic for heart failure, were older and with higher prevalence of previous embolism. This would suggest that, currently, the avoidance of long-term anticoagulation is not considered an indication for a rhythm control strategy in slightly symptomatic patients with criteria for anticoagulation. Although other factors may be contributing to these findings, the suggestion is that such publications may have had a significant influence on our standard clinical practice.

We note a reluctance towards the use of transesophageal echocardiogram (TEE) in the ECV procedure in both of our surveys that had been conducted 7 years apart. The publication of the ACUTE study [10] in 2001 negated the expectations that TEE could reduce embolisms and complications related to anticoagulation, as well as improve the efficacy of ECV because of the shorter time-lapse between the indication and the ECV. Both of our evaluations reflect the few occasions in which this technique may be useful in standard clinical practice. Conversely, we observed almost a complete substitution of monophasic energy (the more frequent option in 2003) for biphasic energy which was used in 2010, and which had been recommended in the 2006 guidelines. The use of biphasic source results in fewer skin lesions and greater rate of reversion to SR for the same amount of energy administered [11,12]. Also, maximum biphasic energy shock is useful, especially in patients with greater body surface area [13]. The slightly higher efficacy of ECV in our 2^nd^ survey of 2010 would be attributable to the use of biphasic energy. However, the more frequent use of biphasic energy and the change in patients’ characteristics did not result in a higher rate of SR at 3 months of follow-up. Biphasic energy could benefit a patient with a profile of higher probability of AF relapse due to higher body mass index [14]. The use of anti-arrhythmic drugs was relatively high and similar in both surveys. Although its use was related to better rates of SR at 3 months of follow-up, its efficacy was relatively low. The introduction of new anti-arrhythmic drugs with higher efficacy and less adverse effects should help to improve these outcomes in the near future. Around half of the patients had been treated with ACE inhibitors/angiotensin receptor blockers (most of them for hypertension), without significant differences between both registries. The benefit of these drugs in the prophylaxis of AF relapse post-ECV has been clear in the published studies [15,16] and this is reflected in the 2001 and 2006 ESC/AHA/ACC guidelines as well as the 2010 ESC guidelines, which do not recommend its use.

Of interest is the change in the number of ECVs performed in tertiary (referral) hospitals *versus* district (general) hospitals. Overall, in the district hospitals we observed a clear decrease in the number of ECVs performed while, in tertiary hospitals, there was an increase. The introduction of catheter ablation may explain these findings, with the use of ECV as a bridge to ablation. As mentioned earlier, this reflects the interest in the rhythm control strategy if there are appropriate therapeutic measures that impact on effective SR maintenance.

Our study has some limitations. One of them is the lack of information on the total number of patients with AF attended-to in our clinics. In consequence, we cannot strictly affirm that there has been a decrease in the proportion of patients treated with rhythm *vs.* rate control strategy. However, if we assess the increasing prevalence of AF [17,18] related, above all, to aging we may deduce a decrease in the proportion of patients with AF treated with the rhythm control strategy; the proportion probably being higher than that indicated by the decrease in the number of ECV that we observed. In our population the mean age increased from 45.8 to 47 years and the number of people >60 years of age increased by 14% during this period. Another limitation of our study is the lack of information on symptoms strictly related to AF. Symptoms described in our study only related to heart failure. A scale of symptoms related to AF had been proposed in 2007 by the EHRA [19] and was included in the 2010 ESC guidelines. As such, it had not been available at the time of our first survey performed in 2003. However, we believe that the attempt to relieve, or improve, symptoms of heart failure may be considered when an ECV is indicated, as had been assessed in our survey.

## Conclusions

In conclusion, we observed that ECV technique continues to be used widely as treatment for patients with AF; albeit applied to candidate patients with different characteristics.

## Abbreviations

ECV, Electrical cardioversion; AF, Atrial fibrillation; SR, Sinus rhythm.

## Competing interest

None

## Authors’ contributions

JMA: conception and design, analysis and interpretation of data, drafting the manuscript. XV: conception and design, acquisition of data. CR: conception and design, acquisition of data. SP: acquisition of data. RV: acquisition of data. NC: acquisition of data. JPR: acquisition of data. XS: acquisition of data. All authors read and approved the final manuscript.

## Funding

This work was supported by the Catalan Society of Cardiology and funded, in part, by a non-directed grant from Sanofi-Aventis. The funding bodies had no involvement in the generation of the data, or their interpretation, or in the decision to publish.

## Pre-publication history

The pre-publication history for this paper can be accessed here:

http://www.biomedcentral.com/1471-2261/12/42/prepub
